# Feasibility and usefulness of ultrasonography in idiopathic intracranial hypertension or secondary intracranial hypertension

**DOI:** 10.1186/s12883-016-0594-3

**Published:** 2016-06-02

**Authors:** Piergiorgio Lochner, Francesco Brio, Maria Luisa Zedde, Sandro Sanguigni, Lorenzo Coppo, Raffaele Nardone, Andrea Naldi, Daniele Sola, Erwin Stolz

**Affiliations:** Department of Neurology, University of the Saarland, Homburg Saar, Germany; Department of Translational Medicine, Section of Neurology, University of Piedmont East “A. Avogadro”, Novara, Italy; Department of Neurological, Biomedica, and Movement Sciences, University of Verona, Verona, Italy; Neurology Unit, Stroke Unit, IRCCS, Arcispedale Santa Maria Nuova, Reggio Emilia, Italy; Department of Neurology, General Hospital Madonna del Soccorso, San Benedetto del Tronto, Italy; Department of Neurology, Christian Doppler Klinik, Paracelsus Medical University, Salzburg, Austria; Department of Internal Medicine, Health Sciences, University “A. Avogadro”, Novara, Italy; Neurological Practice, and Department of Neurology, Justus-Liebig-University Giessen, Juergen-Ponto-Platz 2, D-60329 Frankfurt am Main, Germany

**Keywords:** Sonography, Idiopathic intracranial hypertension, Optic nerve

## Abstract

**Background:**

Transorbital sonography (TOS) has been proven to be able to non-invasively detect elevated intracranial pressure. In this condition TOS shows an increase in optic nerve sheath diameter (ONSD). It has been suggested that internal jugular vein valve insufficiency (IJVVI) may represent a factor contributing to the pathogenesis of idiopathic intracranial hypertension (IIH). The aim of this study was to investigate whether patients with IIH or secondary IH have higher ONSD values and higher frequency of IJVVI compared to subjects without IH.

**Methods:**

Twenty-one patients with newly diagnosed IIH or secondary IH were prospectively evaluated and compared with 21 age, gender and BMI-matched controls. Experienced vascular sonographers used B-mode TOS to evaluate ONSD, optic nerve diameter (OND) and IJVVI. CSF opening pressures were also measured.

**Results:**

ONSD values were significantly higher in patients (6.50 ± 0.67) than controls (5.73 ± 0.66; *p* < 0.0001). No differences were found in OND values between patients (2.99 ± 0.26) and controls (2.93 ± 0.41; *p* = 0.574). No correlation was demonstrated between ONSD and CSF opening pressure (*r* = 0,086) (*p* = 0.73). No difference in frequency of IJVVI between patients (11/42 valves, 26 %) and controls (9/42, 21 %) was observed (*p* = 0.777).

**Conclusions:**

Increased ONSD values detected by TOS support the diagnosis of IH. Our results do not support the hypothesis of a venous congestion as a potential factor contributing to the pathogenesis of IIH.

**Trial registration:**

Not applicable. Observational, non-interventional study.

**Electronic supplementary material:**

The online version of this article (doi:10.1186/s12883-016-0594-3) contains supplementary material, which is available to authorized users.

## Background

Intracranial hypertension (IH) may either present as idiopathic intracranial hypertension (IIH), also known as “pseudotumor cerebri syndrome” or arise from an identifiable structural cause.

IIH is a disorder of unknown etiology characterized and defined by an increase of intracranial pressure (ICP) without neuroradiological abnormalities [1–3]. Typical signs and symptoms of IIH consist of headache, pulsatile tinnitus, transient visual obscurations, blurred vision, diplopia, and papilloedema [1].

Secondary IH may be clinically indistinguishable from IIH and results from an identifiable medical condition, medication toxicity, or venous abnormalities leading to elevated ICP [3, 4].

The diagnosis of IH can be challenging if the patient does not present with typical symptoms of IH and papilledema but a with a low cerebrospinal fluid opening pressure (nadir of pressure wave) [3]. The diagnosis can be even more difficult if the patient has typical symptoms but no papilledema or 6th nerve palsy; in this case the diagnosis can only be suspected if some neuroradiological abnormalities indicative of elevated ICP such as empty sella, flattening of the posterior aspect of the globe, distention of the perioptic subarachnoid space with or without a tortuous optic nerve, and transverse venous sinus stenosis are present [3].

In previous studies, transorbital sonography (TOS) has been demonstrated to be a reliable technique for non-invasive detection of elevated ICP in neurocritical care patients [5, 6]. This is possible because the optic nerve sheath communicates with the subarachnoid space and cerebrospinal fluid flows freely between the cranium and orbit within the subarachnoid space. Hence, increased ICP is transmitted to the optic nerve sheath and may be detected by increased size of the ONSD [7].

In clinical practice IIH is a frequent differential diagnosis in patients with chronic headache, especially when they are obese. Therefore, a rapid, non-invasive screening is of obvious advantage. To date, only few studies (mostly case reports or small case series [8–10] have explored the role of TOS in supporting the diagnosis of IIH, but in the literature there are no definite data on the utility of ONSD measurements in IIH and secondary IH. Moreover, one sonographic study showed that patients with IIH have a higher frequency of internal jugular vein valve incompetence (IJVVI) compared with healthy controls [11]. IJVVI has therefore been proposed as a further potential factor contributing to the pathogenesis of increased ICP in IIH [11].

### Objective

The aim of this study was to investigate whether patients with IIH have higher ONSD values and higher frequency of IJVVI compared to controls matched for age, gender, and body mass index (BMI).

## Methods

### Patients and controls

Written informed consent was obtained from all participants before entering this prospective study. The study was approved by the appropriate local ethics committees in Italy (Bolzano) and Germany (University of Giessen) and was performed in accordance with the ethical standards of the 1964 Declaration of Helsinki. Subsequently, study subjects were recruited in the Departments of Neurology in clinics in Saarbrücken (Germany), Merano, San Benedetto del Tronto, Reggio Emilia and Novara (Italy) between May 2014 and February 2015. Patients were diagnosed either with primary or secondary IH according to the current diagnostic criteria [3]. The control group consisted of patients matched for age, sex, and BMI who suffered from neurological disorders not associated with elevated ICP and who had not undergone lumbar puncture in the past.

The controls were recruited in the same hospital as the patients and were examined by the same investigator.

Each participant underwent general medical, ophthalmologic and neurological examination, basic laboratory investigations, and magnetic resonance imaging of the brain. Lumbar puncture with measurement of CSF pressure was performed in IH patients in the left lateral decubitus (recumbent) position with posterior measurement of CSF opening pressure with legs fully extended [3].

### Transorbital sonography

Five experienced investigators (P.L., S.E., S.S., M.L.Z., L.C.) with expertise in venous and transorbital sonography performed the examinations according to previously described protocols [10–15]. All investigators had similar expertise in TOS, being acknowledged as highly qualified sonographers from the German and Italian Societies for Ultrasound in Medicine.

The high reliability of this technique has been reported in previous studies [12–14].

The investigators involved in the present study previously demonstrated high intra- and interrater reliability of TOS, by independently assessing sonographic parameters (ONSD, OND in healthy subjects) [14].

B-mode TOS was performed by three investigators (PL, SE, LC) using an Aplio XG equipped with a PLT-1204AX: 7.2-14 MHz Linear Probe (Toshiba Medical Systems, Nasu, Japan) and by two investigators (MLZ, SS) using an IU 22 equipped with a 15L8 transducer (Philips, Amsterdam, the Netherlands).

Operators were unaware of the condition of patients and all efforts were made to ensure blinding. Participants were instructed not to talk to the operators and operators were not allowed to ask the patients questions.

Subjects were examined in supine position with the upper part of the body and the head elevated to 20–30° in order to avoid any pressure on the eye. Participants were asked to keep their eyes shut in a mid-position of the bulb and to suppress any eye movements.

**Fig. 1:**
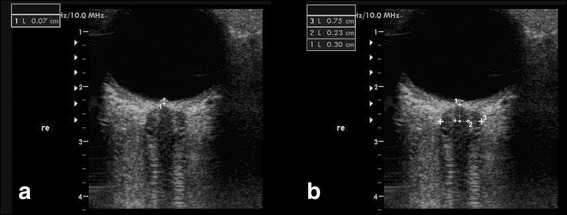
TOS in B mode of the eyeball and the optic nerve in a patient with IH. Panel **a** Optic disc elevation (ODE) is gauged between the fundus and the dome of the papilla in a patient with IH. Panel **b** Optic nerve sheath diameter (ONSD) and optic nerve diameter (OND). ONSD and OND were measured 3 mm behind the papilla (*1*) in an axial plane showing the optic nerve in its longitudinal course. The *dotted lines* denote the OND (*2*) and the ONSD (*3*)

For safety reasons of possible biomechanical side effects, the mechanical index (MI) was reduced to 0.2. The linear array probe (insonation frequencies 7.5 to 10 MHz) was placed on the temporal part of the closed upper eyelid using a thick layer of ultrasound gel. The anterior part of the optic nerve was depicted in an axial plane showing the papilla and the optic nerve in its longitudinal course. ONSD and OND were assessed 3 mm proximal of the papilla. In order to measure the ONSD, the distance between the external borders of the hyperechogenic area surrounding the optic nerve was quantified (Fig. 1 Panel b). The OND was measured marking the internal borders of this formation (Fig. 1, Panel b). In order to minimize intra-observer variability, each bulb was examined three times and the means were calculated. Ultrasound was also used to evaluate the presence of papilledema [9, 10]. The presence of papilledema was assessed as the elevation of the optical disk (ODE) above the level of the retina. Measurements were performed as the distance between the fundus and the dome of the papilla (with the first caliper on the uppermost part of the swollen disc and the second caliper on the strongly reflecting line, which represents the lamina cribrosa) (Fig. 1, Panel a) [9, 10]. The presence of papilledema was defined as ODE values higher than 0.6 mm, according to a recent study showing that an ODE >0.6 mm predicts the presence of fundoscopic optic disc edema with a sensitivity and specificity of 82 and 76 %, whereas a threshold value of 1.0 mm has a sensitivity of 73 % and specificity of 100 % [16].

Finally, TOS was performed before lumbar puncture (Fig. 1, Panel a).

### Extracranial sonography

The evaluation of IJVVI was performed according to previously published protocols [11]. Each patient was asked to place his or her head in a straight position to avoid flow alterations caused by unilateral or bilateral venous outflow obstruction. A large amount of gel was placed on the skin of the patient and great care was taken not to compress the cervical veins when the probe was applied over the neck to obtain reliable velocity measurements. In order to assess IJVVI the patient was asked to perform a Valsava maneuver while a Doppler spectrum was obtained from the vessel above the valve plane Diagnosis of IJVVI was made when the duration of the reflux (reverse flow assessed in the respiratory pause) exceeded 0.88 s [11] (Fig. 4).

### Statistical analyses

Assuming a median diameter of ONSD in controls of 5.0 mm based on published data, the sample size needed to detect a mean difference of 1 mm was estimated to be 10 cases and 10 controls at a level of significance of 0.05 and a beta of 0.20, i.e. a power of 80 %.

A receiver operator characteristics (ROC) curve was also constructed to determine the optimal ONSD cut-off to detect ICP values >25 cm H_2_0.

Values are expressed as mean ± standard deviation if not indicated otherwise.

Differences between groups were examined using *t*-test and chi-square test with statistical significance set at the 0.05 level. Differences between more than two groups were examined using an analysis of variance (ANOVA) followed by the post hoc tests.

All analyses were performed using dedicated statistical software (IBM® Statistical Package for Social Science (SPSS), version 21.0, New York, USA).

## Results

### Demographic characteristics

Demographic characteristics of patients and controls are summarized in Table 1.

**Table 1:** Demographic characteristics of patients with intracranial hypertension (IH) and controls

	IH Patients *N* = 21	Controls *N* = 21	*p*-value
Age (years)	36. ± 10.8	41.7 ± 11.9	0.112
BMI (kg/m^2^)	33.3 ± 2.7	32.3 ± 5.6	0.495
Gender, Females/Males	17/4 (81 %/19 %)	14/7 (67 %/33 %)	0.495
CSF pressure (cmH_2_O)	36.7 ± 11.8	NA	

Values were expressed as mean ± standard deviation. *NA* not applicable

The control group consisted of 21 subjects (14 females and 7 males) aged 41.7 ± 11.9 years and with a BMI of 32.3 ± 5.6 kg/m^2^. These subjects suffered from primary headache (*n* = 5), syncope (*n* = 7), transient ischemic attack (*n* = 3), peripheral neuropathy (*n* = 6). No subject had clinical signs of elevated ICP and had normal neuroradiological findings in computed tomography (6 controls) or in magnetic resonance imaging (15 controls).

The IH group consisted of 21 patients (17 females and 4 males) aged 36.3 ± 10.8 years with a BMI of 33.3 ± 2.7 kg/m^2^. Sixteen patients had idiopathic IH and 5 had secondary IH (4 as a consequence of cerebral venous sinus thrombosis, 1 secondary to a meningioma invading the superior sagittal sinus).

Age, sex and BMI did not significantly differ between cases and controls (Table 1). Details on medication in primary and secondary IH are summarized in Table 2.

**Table 2:** Details of the medication in primary and secondary intracranial hypertension (IH) patients

	Drug
Idiopathic IH	
Patient 1	ethinylestradiol/drospirenone
Patient 2	ethinylestradiol/gestodene
Patient 3	levothyroxine
	folic acid
Patient 4	pantoprazole
Patient 5	colecalciferol
Patient 6	propranolol
Patient 7	amitriptyline
Patients 8 to16	none
Secondary IH	
Patient 17	ranitidine
Patient 18	allopurinol
Patient 19	perindopril
	acetylsalicylic acid
	allopurinol
Patient 20–21	none

### Transorbital sonography

ONSD and OND measurements for both eyes were obtained in 100 % of subjects.

**Fig. 2:**
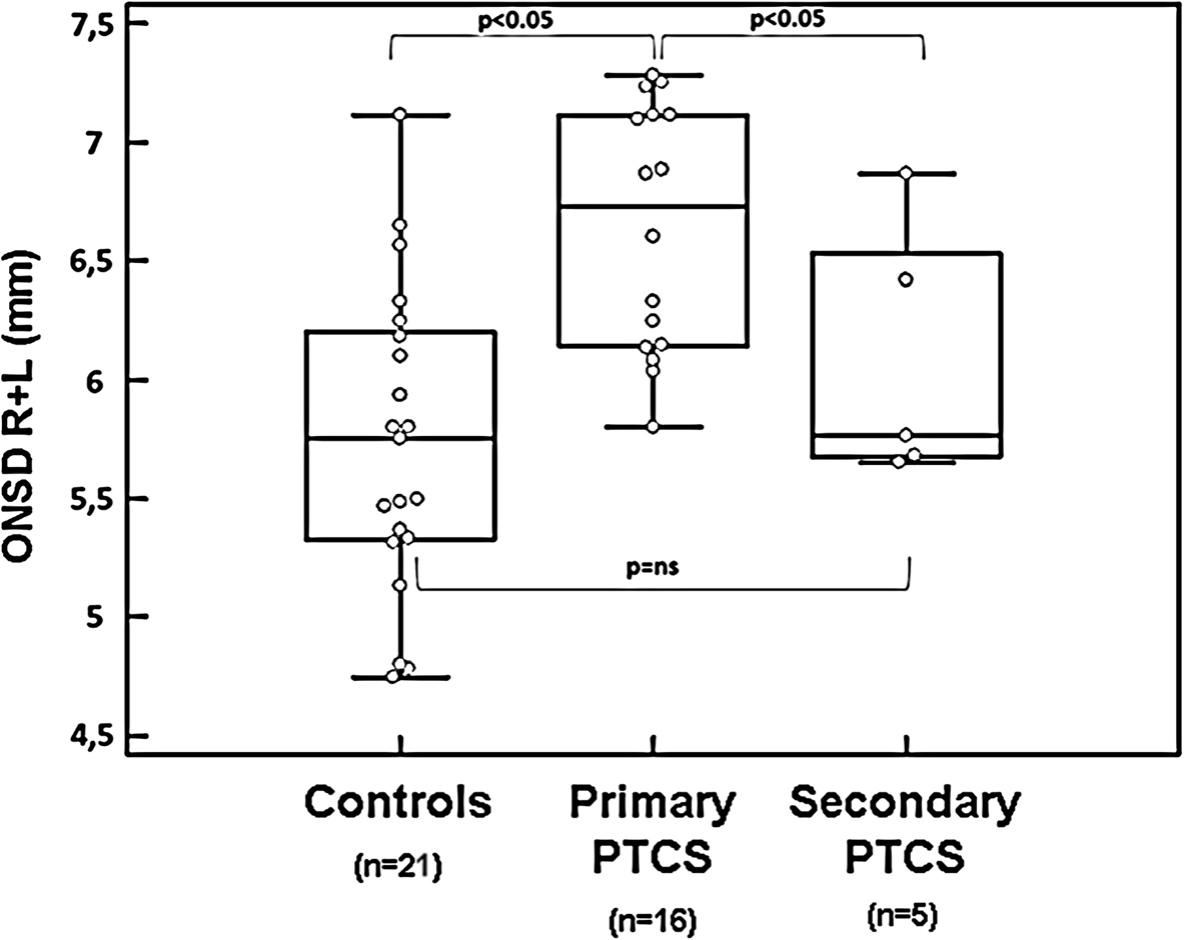
Optic nerve sheath diameter in controls and primary and secondary intracranial hypertension (IH). Intracranial hypertension (IH) may be either primary (idiopathic intracranial hypertension, also known as “pseudotumor cerebri syndrome”, IH) or arise from an identifiable secondary cause. Diagnostic criteria are reported in [3]. *Box plot* indicating median value ± interquartile range in controls and primary and secondary IH

The mean ONSD was significantly enlarged bilaterally among individuals with IH (6.50 ± 0.67 mm) compared to the controls (5.73 ± 0.66 mm) (*p* < 0.001). ONSD mean values were higher in patients with primary (ONSD 6.50 ± 0.67 mm) compared with those with secondary IH (ONSD 6.09 ± 0.57 mm) (*p* = 0.039) (Fig. 2).

No difference in mean OND values were found between patients and controls (*p* = 0.574). No differences were found in mean OND values between primary (OND 2.99 ± 0.26 mm) and secondary IH (OND 3.02 ± 0.39 mm) (*p* = 0.771).

A bilateral papilledema (ODE) was found in 20 of the 21 patients in the IH group (95 %), and in none of the controls. In the patient without papilledema a diagnosis of idiopathic IH could be made because of unilateral 6th nerve palsy, increased opening cerebrospinal pressure and normal neuroimaging.

Mean ODE in IH patients was 0.8 ± 0.43 mm on the right side and 0.8 ± 0.38 mm on the left side (*p* = 1).

More details on ONSD, OND and the presence of papilledema are given in Table 3.

**Table 3:** Optic nerve sheath diameter (ONSD), optic nerve diameter (OND), and optic disc elevation (ODE) values in patients with intracranial hypertension (IH) and in controls. None of the controls had papilledema (i.e. ODE = 0 mm)

	IH Patients *N* = 21	Controls *N* = 21	*p*-value
Mean ONSD (mm)	6.5 ± 0.67	5.73 ± 0.66	<0.0001
Right ONSD (mm)	6.5 ± 0.65	5.7 ± 0.7	
Left ONSD (mm)	6.4 ± 0.69	5.8 ± 0.7	
Mean OND (mm)	2.99 ± 0.26	2.93 ± 0.41	0.574
Right OND (mm)	2.99 ± 0.27	3.02 ± 0.35	
Left OND (mm)	2.99 ± 0.24	2.98 ± 0.34	
Mean ODE (mm)			
Right ODE (mm)	0.8 ± 0.43		
Left ODE (mm)	0.8 ± 0.38		

There were no correlations between ONSD and age, gender, or BMI neither in patients with IH nor in controls. No correlation was demonstrated between ONSD and CSF opening pressure (*r* = 0.086, *p* = 0.73). In the IH group, a positive association between CSF opening pressure and BMI was found (*r* = 0.84, *p* = 0.003).

**Fig. 3:**
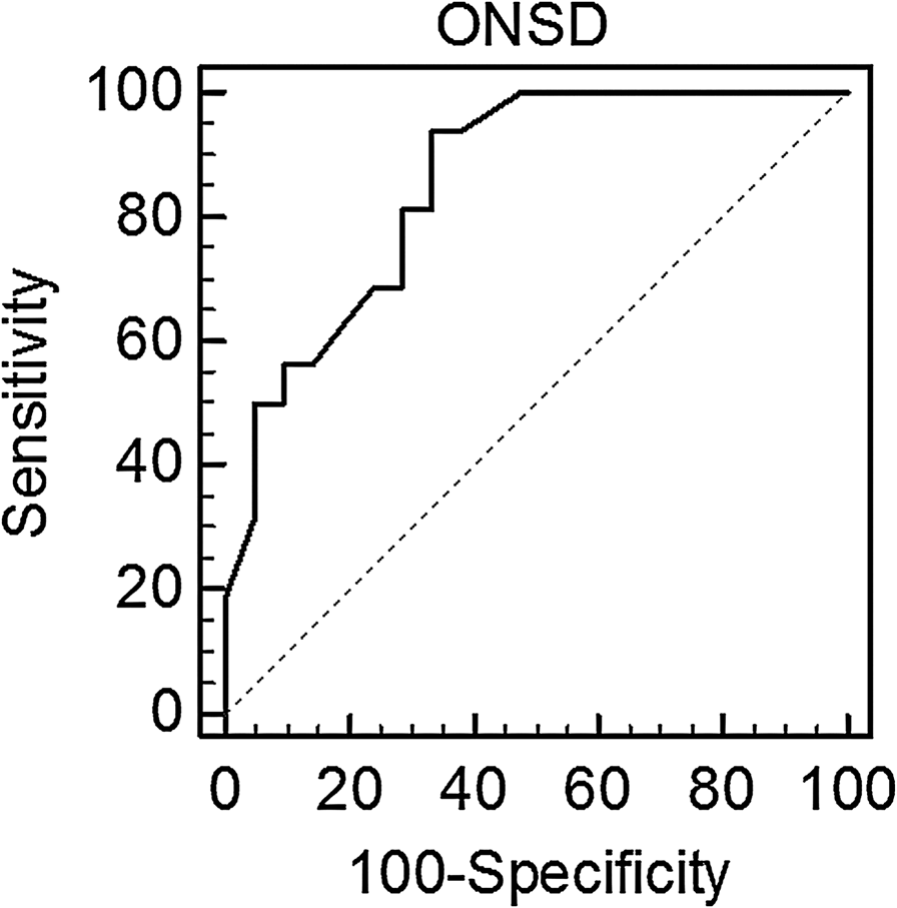
Reciever Operator Curve (ROC) for optic nerve sheath diameter (ONSD) given in [mm] related to intracranial pressure (ICP). Raised ICP is defined as an ICP >25 cmH_2_O. ONSD accurately predicted an elevated ICP (AUC = 0.85; 95 %; CI 0.68 to 0.94; *p* = 0.0001). *AUC* area under the curve, *CI* confidence interval

The ROC curve analysis revealed that the optimal cut-off value of ONSD for predicting elevated intracranial pressure was 5.93 mm (AUC = 0.85; 95 % confidence intervals: 0.68 to 0.94 mm, *p* = 0.0001). Adopting this cut-off value, the sensitivity and specificity of this cut-off value were 93 and 67 %, respectively (Fig. 3).

### Internal jugular vein valve incompetence

**Fig. 4:**
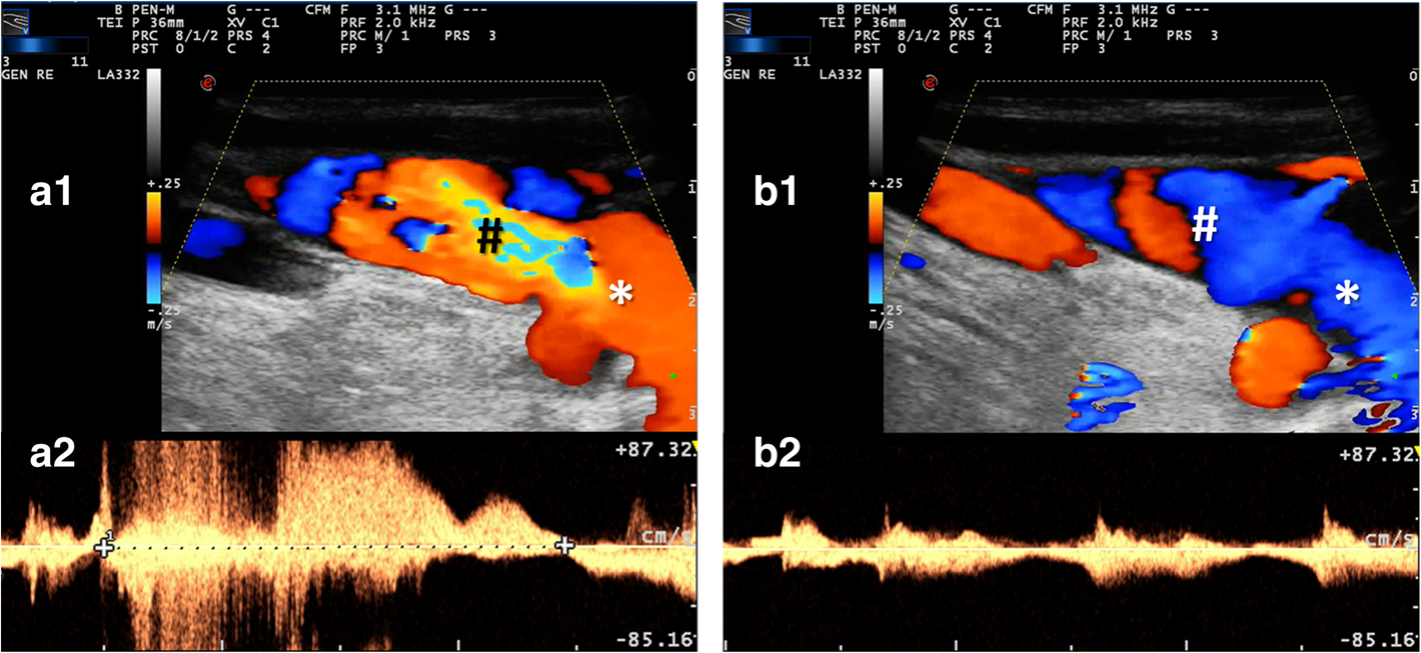
Internal jugular vein valve insufficiency (IJVVI) and a normal jugular flow waveform. **a1**-**a2** IJV flow through the IJV leaflets at the base of the neck during Valsalva maneuver. **b1**-**b2** IJV flow through the IJV leaflets at the base of the neck during normal breathing cycles. a1 and b1 show the Colour-mode appearance of the IJV flow in a longitudinal scanning plane starting from the jugular valve (*), respectively during Valsalva maneuver and normal breathing. The sample volume (#) is placed just after the jugular valve and the corresponding flow waveform is a2 and b2, respectively during Valsalva maneuver and during normal breathing. In a2 a retrograde flow lasting about 2 s (starting with > and ending with <) is shown. In b2 a normal jugular flow waveform is imaged with heart and breath modulation

Internal jugular vein valve insufficiency was found in 11 out of 42 valves in patients with IH and in 9 out of 42 valves in controls (*p* = 0.78) (Fig. 4). Anonymized study data on which the results have been based are included as additional material (Additional file 1).

## Discussion

In this prospective study we analysed ONSD, OND, ODE, and IJVVI in adults with newly diagnosed IH versus controls. The main finding of our study is that ONSD values of patients with IH were significantly higher than in controls. This result is consistent with a previous A-mode sonographic study showing that 47 % of patients with elevated ICP had significantly increased ONSD values [17]. Furthermore, our data are in line with those from a previous B-scan echography study which analyzed ONSD, OND, and ODE in adults with newly diagnosed IH both before and after lumbar puncture [10].

Interestingly, ONSD values in our subgroup of 16 patients with idiopathic IH (6.65 ± 0.63 mm) are very similar to those from that previous study (6.4 ± 0.6 mm) [9, 10]. This finding supports the high reliability and reproducibility of TOS in detecting raised intracranial pressure, as already demonstrated in previous studies comparing simultaneous measurements of sonographic ONSD and intraparenchymal ICP values [5, 6].

We have no clear explanation for the higher ONSD values found in patients with idiopathic IH compared to patients with secondary IH. Considering the very small number of patients in the secondary IH group, such a difference might be simply due to chance alone or to an intrinsic variability of ONSD values across subjects [18]. However, this finding may prompt further studies to investigate whether a difference in ONSD values actually exists between primary and secondary IH.

Our results suggest that B-mode sonographic measurement of the ONSD is a promising method to assess the elevated ICP in patients with IH. More specifically, our data suggest that ONSD values higher than 5.93 mm support the diagnosis of increased ICP with high sensitivity (93 %) and relatively high specificity (67 %).

No difference was found in OND values between patients with IH and controls, suggesting that OND has a poor correlation with ICP, as previously demonstrated [19]. The measurement of OND values seems therefore to have limited clinical value in the diagnosis of IH. The explanation is that not the optic nerve itself but rather the subarachnoid space around the optic nerve is enlarged when ICP increases.

Papilledema is a delayed consequence of chronic CSF accumulation in the retrobulbar optic nerve sheath due to raised pressure in CSF in cranial cavity [20]. Each condition leading to increased ICP compresses the optic nerve, causing stasis of axonal transport with subsequent swelling of the ON axons, which may result in a vision-threatening ophthalmologic condition [20]. We found a bilateral papilledema in 95 % of patients with IH; although this sign requires a few days to develop. It represents a very useful clinical sign in patients where a chronic elevation of ICP is suspected. Furthermore, this finding emphasizes the importance of prompt recognition and treatment of this condition to reduce and avoid the risk of loss of vision.

Our results do not support the hypothesis of a venous congestion as a potential factor contributing to the pathogenesis of idiopathic IH [11]. In contrast to a previous study [11], we found no difference in IJVVI between patients with IH and controls. In this regard, it is possible that -our study underpowered to detect a difference in the frequencies of IJVVI. However, this result sheds doubts on a possible relationship between IJVVI and increased ICP.

Our study has some limitations. First, we included a relatively small number of patients (*n* = 21), particularly with secondary IH (only 6). We nonetheless plan to conduct a further study focusing on secondary IH. Second, this was a multicenter study with several sonographers performing the TOS. However, previous studies have shown that TOS with ONSD and OND measurements have excellent intra- and interobserver reliability [12–14] and a preliminary assessment of intra-and interrater reliability across the five sonographers involved in the present study showed negligible discrepancies between ultrasonographic parameters. Third, we did not perform an ultrasound study of intracranial venous circulation. A relatively high prevalence of stenosis of the transverse sinuses has been reported in patients with IH. However, whether this stenosis represents the cause or is rather a consequence of IH is still unclear [21]. Fourth, we did not perform lumbar puncture in controls. However, none of them had neurological disorders associated with increased ICP values or clinical signs suggestive of IH. Finally, for practical reasons we were not able to randomize the operator sequence.

## Conclusions

In conclusion, our study shows that sonographic measurements of ONSD values may be useful in detecting raised ICP in patients with presumed IH. In these patients values of ONSD higher than 5.93 mm support the diagnosis of IH with high sensitivity (93 %) and relatively high specificity (67 %). The role of IJVVI and stenosis of the transverse sinuses in the pathogenesis of idiopathic IH doubtful and is not supported by our findings. Further studies are required to answer this point conclusively.

### Ethics approval and consent to participate

Written informed consent was obtained from all participants before entering this prospective study. The study was approved by the appropriate local ethics committees in Italy Italy (Bolzano 12/2014) and Germany (University of Giessen 10604) and was performed in accordance with the ethical standards of the 1964 Declaration of Helsinki. Subsequently, study subjects were recruited in the Departments of Neurology in clinics in Saarbrücken (Germany), Merano, San Benedetto del Tronto, Reggio Emilia and Novara (Italy) between May 2014 and February 2015.

### Consent for publication

Not applicable. No individual patient data have been reported in this study.

### Availability of data and materials

The dataset supporting the conclusions of this article is included within the article an anonymous supplementary spreadsheet file.

## Additional file

Additional file 1: Anonymized Data. (XLSX 109 kb)

## Abbreviations

BMI: body mass index
CSF: cerebral spinal pressure
ICP: intracranial pressure
IH: intracranial hypertension
IJVVI: internal jugular vein valve incompetence
ODE: optic disc elevation
OND: optic nerve diameter
ONSD: optic nerve sheath diameter
TOS: transorbital sonography
