# Erratum to: Feasibility and usefulness of ultrasonography in idiopathic intracranial hypertension or secondary intracranial hypertension

**DOI:** 10.1186/s12883-016-0615-2

**Published:** 2016-07-01

**Authors:** Piergiorgio Lochner, Francesco Brigo, Maria Luisa Zedde, Sandro Sanguigni, Lorenzo Coppo, Raffaele Nardone, Andrea Naldi, Daniele Sola, Erwin Stolz

**Affiliations:** Department of Neurology, University of the Saarland, Homburg, Saar Germany; Department of Translational Medicine, Section of Neurology, University of Piedmont East “A. Avogadro”, Novara, Italy; Department of Neurosciences, Biomedicine and Movement Sciences, University of Verona, Verona, Italy; Neurology Unit, Stroke Unit, IRCCS, Arcispedale Santa Maria Nuova, Reggio Emilia, Italy; Department of Neurology, General Hospital Madonna del Soccorso, San Benedetto del Tronto, Italy; Department of Neurology, Christian Doppler Klinik, Paracelsus Medical University, Salzburg, Austria; Department of Internal Medicine, Health Sciences, University “A. Avogadro”, Novara, Italy; Neurological Practice, and Department of Neurology, Justus-Liebig-University Giessen, Juergen-Ponto-Platz 2, Frankfurt am Main, D-60329 Germany

## Erratum

After publication of the original article [1], it came to the authors’ attention that there were errors in the author list and the affiliations. Francesco Brigo’s surname had been spelled incorrectly, and the department of his affiliation (University of Verona) had been incorrectly specified. These errors have been corrected in the original article.
